# Reducing Salt Intake and Exercising Regularly: Implications From Molecular Dynamics Simulations of Endothelial Glycocalyx

**DOI:** 10.3389/fphys.2018.01667

**Published:** 2018-11-21

**Authors:** Xi Zhuo Jiang, Kai H. Luo, Yiannis Ventikos

**Affiliations:** Department of Mechanical Engineering, University College London, London, United Kingdom

**Keywords:** endothelial glycocalyx layer, lifestyle, molecular dynamics, ion transport, sodium intake

## Abstract

It is widely accepted that salt intake reduction and regular exercise is a healthy lifestyle, which can prevent cardiovascular diseases (CVD). Meanwhile, there is evidence that the endothelial glycocalyx layer (EGL) is related to CVD. However, how such a healthy lifestyle helps to prevent CVD via the function of the EGL has not been scientifically established. In this research, a series of large-scale molecular dynamics simulations have been conducted to study ion transport inside the EGL under varying flow velocities. Results show that a fast blood flow velocity favors the Na^+^ transport out of the EGL, which can explain the increase in the thickness of an exclusion layer between red blood cells and the EGL under fast blood flow situations, as witnessed in some previous experiments. Based on findings from this fundamental research, a theory is proposed, which can answer the open-ended question “Why do we need to reduce salt intake and exercise regularly”. The findings may also have implications for other therapies to combat cardiovascular diseases.

## Introduction

The endothelial glycocalyx layer (EGL), the first and foremost barrier in direct contact with blood (Curry and Adamson, 2012), is closely related to cardiovascular diseases (CVD) (Rabelink and de Zeeuw, 2015). EGL also acts as a crucial buffer barrier for sodium (Oberleithner, 2012). In the meantime, empirical studies suggest that reducing salt intake (He and MacGregor, 2011, 2018; Aburto et al., 2013) and exercising regularly (Agarwal, 2012; Schuler et al., 2013) can lower the risk of CVD or reduce the chance of CVD getting worse.

When sodium intake exceeds the excretory capacity of the kidney, sodium is retained. Previous studies have experimentally demonstrated that salt overload stiffens vascular endothelial cells and increases vascular sodium permeability, thereby damaging the glycocalyx sodium barrier of vascular endothelium (Oberleithner et al., 2011). Thus, intake of sodium chloride would have implications for the function of the endothelial glycocalyx as a foremost sodium barrier. These findings elucidate the significance of the EGL as a barrier from the perspective of cell mechanics. Also, the EGL features highly negatively charged sugar chains (Weinbaum et al., 2007). How the crucial function of EGL is affected by electrostatics requires further investigation.

In the meanwhile, an interesting phenomenon occurring between the EGL and red blood cells (RBCs) may explain the benefit of doing regular exercise (Lott et al., 2001): when RBCs flow through our vessels, an exclusion layer between the RBCs and EGL forms, facilitating the motion of the RBCs; the layer thickness increases as RBCs accelerate (Vink and Duling, 1996). The behavior of the RBCs on the EGL can be compared to human skiing, and a lubrication theory for the principle of skiing has been extended to understand the formation of the exclusive layer (Feng and Weinbaum, 2000). However, the theory is unable to explain the layer thickness variation with the varying blood velocity. Meanwhile, the charge properties of the EGL have not been considered either.

To scientifically explain the different experimental results and empirical observations, we conduct an *in silico* numerical experiment focusing on Na^+^ ion transport in the presence of the EGL under varying blood flow velocities. The Na^+^ distributions in various scenarios are determined by molecular dynamics (MD) simulations. Based on the results, the function of the EGL as a sodium barrier is discussed from the perspective of electrostatics. Also, explanations will be provided to elucidate the response of the exclusion layer thickness to the varying blood velocities.

## Materials and Methods

### System Construction

**FIGURE 1:**
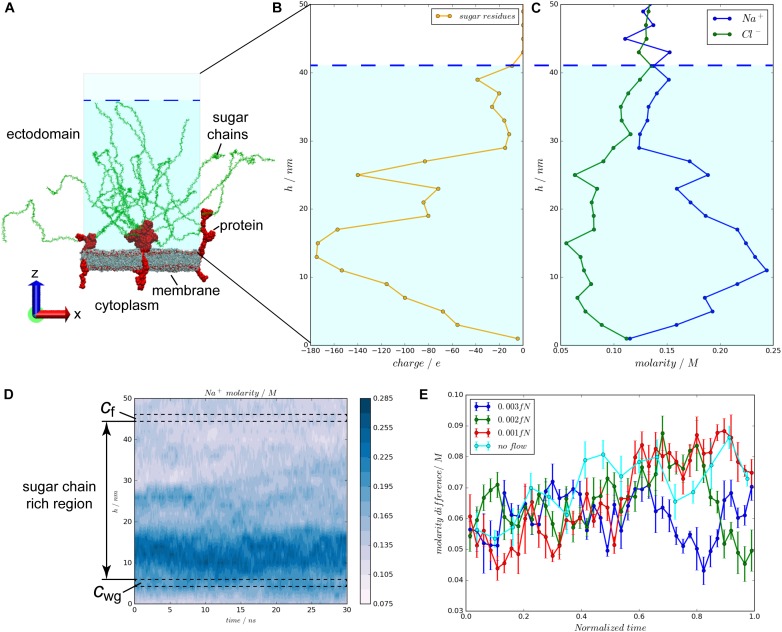
The configuration of the flow/glycocalyx system and charge distributions over the glycocalyx layer under varying flow velocities. **(A)** The configuration of the flow/glycocalyx system. The system is solvated in a NaCl solution with a molarity of 0.1 M. Water and ions are not shown. **(B)** Charge distributions of the negatively charged sugar chains along the height in the ectodomain. **(C)** Molarity distributions of the Na^+^ and Cl^-^ ions along the height in the ectodomain. **(D)** Molarity difference continues throughout the entire simulation. c_wg_ is continuously greater than c_f_ in the simulation. **(E)** The molarity difference between two regions beneath and above the sugar chain region, c_wg_–c_f_, changes with the varying blood flow velocities. The largest average molarity difference appears in the no flow case, followed by the 0.001, 0.002, and 0.003 fN cases in sequence.

A flow/glycocalyx system was constructed, using the currently most detailed structural information of the glycocalyx to mimic flow on a patch of an endothelial cell lipid member (Figure 1A). In this system, three glycocalyx elements are involved. One glycocalyx element is composed of a core protein and six sugar chains. Syndecan-4 (Syn-4) proteoglycan and heparin sulfate (HS) sugar residues were selected to model the glycocalyx core protein and sugar chains, respectively. As shown in Figure 1A, the whole space is divided into two compartments by the lipid bilayer. Above the lipid bilayer is the ectodomain, representing the space outside the endothelial cells, where flow passes by. This region contains negatively charged HS sugar chains, Syn-4 ectodomain in connection with HS sugar chains, water molecules and ions. Below the lipid bilayer is the cytoplasm, representing the inner space of the cell, which is filled with the Syn-4 cytoplasmic protein, water molecules and ions. All the biomolecules are solvated and ionized to 0.1 M NaCl solution. The simulation box is a hexagonal prism with an area of 820 nm^2^ and height of 72 nm. The glycocalyx-flow system comprises about 5,800,000 atoms in total.

### Protocol Details

The TIP3P water model (Jorgensen et al., 1983) was adopted for water molecules. The CHARMM biomolecular force field (MacKerell et al., 1998) was applied on proteins and the lipid bilayer. Force field parameters for sugar residues have been validated in previous studies (Cruz-Chu et al., 2014).

The system was equilibrated under isothermal-isobaric (NPT) and canonical (NVT) ensembles. The velocity Verlet integration method (Allen and Tildesley, 1987) was used to advance the positions and velocities of atoms in time steps of 2 fs. Particle mesh Ewald (Darden et al., 1993) electrostatics with a grid density of 1/Å^3^ was used. The SETTLE algorithm (Miyamoto and Kollman, 1992) was used to enable the rigid bonds connected to all hydrogen atoms. The van der Waals interactions were calculated using a cut-off of 12 Å with a switching function starting at 10 Å. In flow simulations, the Lowe-Andersen thermostat, a specific thermostat exclusively for flow problems, was selected to maintain the temperature at 310 K.

All MD simulations were performed using the software suite NAMD 2.9 (Phillips et al., 2005). The visualization of the molecular structures was performed via the VMD (Humphrey et al., 1996) package. All parallel simulations and non-visualized post-processing were conducted on ARCHER, UK’s national supercomputing service. To obtain a simulation result with physical time of 1 ns, 9,000 compute cores have been simultaneously employed for about 2 h.

Details about the construction of the flow/glycocalyx system and the protocol information can be found in our previous publications (Jiang et al., 2017).

### Flow Simulation and Case Set-Up

To mimic flow, external forces were imposed on oxygen atoms of the water molecules in the ectodomain, which has been successfully demonstrated in previous studies (Jiang et al., 2017, 2018a,b; Pikoula et al., 2018). As reported in a previous study (Jiang et al., 2017), an external force with an order of magnitude of 0.001 fN would generate a laminar flow with a physiological bulk flow velocity; the complex and moving structures of the glycocalyx structures disturb the flow profiles, resulting in an oscillating velocity distribution in space. According to the Newton’s Law of Motion, the resulting bulk flow velocity is assumed to be in proportion to the external force. By imposing various external forces (0.003, 0.002, and 0.001 fN, respectively) on water oxygens in the ectodomain, the changing blood flow velocity conditions were mimicked. A diffusion (no flow) case was included for comparison, in which all the external forces imposed on water oxygens were set to 0.

### Geometric Stratification

To investigate the ion distribution in space, the height origin of the ectodomain was first determined by the average positions of heavy atoms (i.e., carbon, nitrogen, and phosphor atoms) of the upper lipid heads. An ectodomain space with 50 nm in height starting from the origin was divided into 25 equal bins where the charges of sugar chains and the concentrations of ions were individually calculated. Detailed description about the geometric stratification can be found in a previous study (Jiang et al., 2017).

### Statistical Information

The error bars on all bar charts show the standard errors. Statistical significance was determined with *t*-tests.

## Results

The EGL features highly negatively charged sugar chains (Rabelink and de Zeeuw, 2015). As a result, in the initial configuration of the system, Na^+^ ions (Figure 1C) follow a nearly symmetric distribution with the charge distribution of the sugar chains (Figure 1B). Meanwhile, the Na^+^ molarity near the membrane (a space from 2 nm to 12 nm in height) is greater than its counterpart in the flow region (a space from 42 to 50 nm in height). The molarity difference is also ubiquitously found throughout a 30-ns simulation of the flow case with the external of 0.003 fN as shown in Figure 1D. The molarity gradient suggests Na^+^ ion transport through the sugar-chain-rich region. To measure the Na^+^ movement through the sugar-chain-rich region, molarity difference between two 2-nm layers beneath and above (i.e., *c*_wg_ and *c*_f_ in Figure 1D) the sugar-chain-rich region is scrutinized. The time evolutions of the molarity differences, *c*_wg_–*c*_f_, under varying blood flow velocities are compared in Figure 1E. The mean molarity differences over time in the four situations are then calculated. Statistics have shown that the largest mean molarity difference occurs in the no-flow case with the mean difference of (0.0705 ± 0.0012) M, followed by the 0.001 fN case of (0.0670 ± 0.0010) M and the 0.002 fN case of (0.0653 ± 0.0008) M. The smallest average molarity difference occurs in the fastest flow case (0.003 fN) with a mean difference of (0.0620 ± 0.0005) M [*p*(no flow > 0.001 fN) = 0.014, *p*(no flow > 0.002 fN) = 0.0003, *p*(no flow > 0.003 fN) = 2 × 10^-9^, *p*(0.001 fN > 0.002 fN) = 0.09, *p*(0.001 fN > 0.003 fN) = 6 × 10^-6^, and *p*(0.002 fN > 0.003 fN) = 0.0002 by one-side *t*-tests]. Such a sequence suggests that the flow promotes the Na^+^ ion transport across the glycocalyx layer to the main flow region.

## Discussion

It is important to put the reported findings in the context of the existing literature. Previous studies have demonstrated the significance of the EGL as a sodium barrier (Oberleithner, 2012) from the perspective of cell mechanics as mentioned in the Introduction. By introducing the RBCs, the present research further demonstrates the importance of such a barrier in regulating the motion of RBCs, from the perspective of electrostatics. The formation of the repulsive layer between the RBCs and the EGL can be attributed to their repulsive interactions, as the surfaces of the RBCs are coated with negative charges. Even in healthy conditions, if extra salt is taken in, Na^+^ may gather around the EGL and neutralize some of negative charges of the EGL, resulting in a decrease in repulsive interactions between RBCs and EGL and a pertinent decline in the exclusion layer thickness. In other words, sodium renders the endothelial cells “sticky” for RBCs, as observed in a recent experiment (Oberleithner et al., 2015). In this regard, to keep a low level of salt intake can benefit the smooth movement of the RBCs, which has also been validated by experiments (Oberleithner, 2015).

According to the present results, a slightly faster blood velocity assists the transport of Na^+^ ions to the outside of the EGL. Particularly, the number of Na^+^ ions remaining in the EGL decreases, and the net negative charges in the EGL increase. Consequently, the repulsive interaction between RBCs and EGL is strengthened, leading to the thickness growth of the exclusion layer. The thickness increase then facilitates the smooth flowing of the RBCs which are intimately bound with metabolism. It is therefore expected that regular exercise is beneficial for maintaining the normal operation of the body, as the blood velocity will slightly increase during exercise (Lott et al., 2001).

The present research provides an alternative answer to the open-ended question “Why do we need to reduce salt intake and exercise regularly” based on the atomistic behavior of Na^+^ ions under varying blood flow velocities. Indeed, the atomic behavior revealed in this study may also provide scientific evidence for seeking breakthrough in therapies to EGL-related diseases. In future research, dynamics of the EGL in abnormal conditions (e.g., with the shedding of sugar chains) under varying blood flow velocities will be investigated.

## Author Contributions

XJ conducted the simulation and post-processing. KL and YV are co-leaders of the project. All authors contributed to technical discussions, data analysis, and editing of the manuscript.

## Conflict of Interest Statement

The authors declare that the research was conducted in the absence of any commercial or financial relationships that could be construed as a potential conflict of interest.
